# The Impact of Silver Nanoparticles Produced by *Bacillus pumilus* As Antimicrobial and Nematicide

**DOI:** 10.3389/fmicb.2016.01746

**Published:** 2016-11-10

**Authors:** Wael M. Mahmoud, Tamer S. Abdelmoneim, Ahmed M. Elazzazy

**Affiliations:** ^1^Medical Genetics Department, Faculty of Medicine, University of JeddahJeddah, Saudi Arabia; ^2^Biology Department, Faculty of Science, University of JeddahJeddah, Saudi Arabia; ^3^Department of Agricultural Botany, Faculty of Agriculture, Suez Canal UniversityIsmailia, Egypt; ^4^Chemistry of Natural and Microbial Products Department, National Research CentreGiza, Egypt

**Keywords:** silver, nanomaterials, bactericidal, nematicidal, ecofriendly materials

## Abstract

This study evaluates the potential application of silver nanoparticles (AgNPs) as antimicrobial or nematicidal agents produced by the extremophile *Bacillus pumilus*, which was isolated from the alkaline Wadi El-Natrun Lake in Egypt. The AgNPs were characterized by ultraviolet–visible absorption spectroscopy, transmission electron microscopy, and energy dispersive x-ray spectroscopy. The size of AgNPs formed ranged from 20.12 to 29.48 nm. *Panagrellus redivivus* was exposed to different concentrations (0, 50, 100, 150, and 200 μg/mL) of AgNPs in a 5 mL nematode suspension (1 × 10^3^ mL^−1^). The best result occurred at AgNP concentrations of 150 and 200 μg/mL, with death rates of 80 and 91%, respectively, following 48 h of exposure. AgNPs also exhibited potent antimicrobial properties when using Gram-negative and Gram-positive human pathogens, with MIC and MBC values of 5 and 10 μg/mL, respectively. These laboratory assays prove that biologically synthesized AgNPs are an ecofriendly material that can be used in lieu of solvents or toxic chemicals.

## Introduction

Nanosilver, a therapeutically potent molecule, has captivated scientists from various disciplines (Galdiero et al., 2011). Diverse methods are currently being used for nanoparticle production, including physical, chemical, and hybrid systems (e.g., chemical reduction in aqueous or non-aqueous solutions (Petit et al., 1993), sonochemistry (Pol et al., 2002), microemulsions (Solanki and Murthy, 2010), and microwave-based systems Li et al., 2010). However, despite being both robust and technically feasible, many of these techniques involve perilous chemicals, are power intensive, and produce undesirable and/or wasteful byproducts (Kowshik et al., 2003; Mukunthan et al., 2011). Moreover, contamination of nanoparticles may have undesirable downstream effects, especially during therapeutic interventions (Jain et al., 2010). Therefore, there is a pressing need for harmless and effective nanoparticle production systems.

Over the last few years scientists have made significant progress in synthesizing ecofriendly and economically innocuous nanoparticles from different microorganisms (e.g., bacteria, actinomycetes; Ahmad et al., 2003; Sadowski et al., 2008; Sintubin et al., 2009). Biosynthesis of silver nanoparticles has been reported from bacteria, fungi, yeast, plants, and fruits (Jha et al., 2009). Gold and platinum nanoparticles have been widely utilized as novel antiviral, antimicrobial, anticancer, and anti-inflammatory agents (Hu et al., 2006; Jain et al., 2008). The potent antimicrobial characteristics of AgNPs have contributed to their use in a variety of medical and ecological applications, as well as in various consumer products (Kumar et al., 2008; Chaudhari et al., 2012).

*Panagrellus redivivus* is a free living nematode that plays an important role in decomposition, nutrient cycling, and ecosystem distribution in various microbial habitats (Félix and Braendle, 2010; Kim et al., 2012). *Caenorhabditis elegans* and *P. redivivus* are generally the preferred test nematodes in nanotoxicology (Kim et al., 2008, 2012; Roh et al., 2009; Wang et al., 2009). The toxicity of AgNPs toward nematodes has been previously studied in the gut and digestive organs of *C. elegans* (Meyer et al., 2010; Mohan et al., 2010). Here, we focused on the biosynthesis of AgNPs using the extracellular extract of a thermophilic strain, *Bacillus pumilus*, evaluating its use as an antimicrobial against various human pathogens and assessing the nematicidal effect toward *P. redivivus*. We also characterized the biosynthesized AgNPs using UV-visible spectroscopy, transmission electron microscopy, Fourier transform infrared spectroscopy, and energy dispersive X-ray spectroscopy.

## Materials and methods

### Bacterial strain and sample preparation

The bacterial strain used in this study was isolated from the alkaline Wadi El-Natrun Lake in Egypt. The soil samples were collected in sterilized plastic bags and transferred to the laboratory. One gram of each soil sample was serially diluted, 100 μL of each dilution was poured and spread onto nutrient agar plates, and the plates were incubated at 60 ± 2°C for 48 h, distinct morphological colonies were picked and purified by serial streaking on nutrient agar (NA) plates. 16S rRNA gene sequencing was used to identify the bacterial species (Holt et al., 2000; Murray et al., 2005).

### Biomass production

The bacterial isolates were cultured in alkaline nutrient broth medium to produce biomass for silver biosynthesis. The culture flasks were incubated for 24 h and agitated on an orbital shaker at 220 rpm at pH 10 and 60°C. The bacterial biomass was harvested by centrifugation at 5000 rpm for 10 min after 24 h growth. The biomass was discarded and the supernatant material was collected for further processing.

### Synthesis of silver nanoparticles (AgNPs)

AgNO_3_ (1 mM) was added to the culture supernatant and allowed to react for 24 h; a control without silver ions was also run. All of the bacterial strains were screened for their ability to synthesize AgNPs by observing visible color changes and analyzing samples by UV-visible spectroscopy. Only the strains capable of AgNP synthesis were selected for further characterization of antimicrobial activity and by Fourier transform infrared spectroscopy, transmission electron microscopy, and energy dispersive X-ray spectroscopy.

### Characterization of silver nanoparticles

#### UV-visible spectroscopy

Screening for AgNP synthesis was performed via visual observation of color conversion of the culture filtrate and UV-visible spectroscopy (Labomed-Korea; 200–1000 nm).

#### Fourier transform infrared (FTIR)

The FTIR spectrum of a dried sample was recorded on a Perkin Elmer instrument ranging from 450 to 4000 cm^−1^ at a resolution of 4 cm^−1^.

#### Energy dispersive X-ray spectroscopy (EDX)

Elemental analysis was performed at 20 keV. A fine film of the sample was prepared on aluminum foil (1 × 1 cm) by placing 100 μL of the supernatant onto the foil sheet and allowing it to dry for 30 min.

#### Transmission electron microscopy (TEM)

Samples were prepared by removing the silver nanoparticles from the extract by centrifugation (5000 rpm for 15 min) and washing (twice) in sterile distilled water. A drop of the sample was applied to a carbon-coated copper grid. After about 1 min, the excess solution was removed using blotting paper and the grid was air-dried before analysis.

#### *In-vitro* antibacterial assay of silver nanoparticles by agar-well diffusion assay

The well-diffusion technique was used to test the antimicrobial capabilities of the synthesized silver nanoparticles against multiple pathogenic strains, including: Methicillin-resistant Staphylococcus aureus [ATCC 43330 (MRSA)], *Staphylococcus epidermidis* [ATCC 12228 (*S. epidermidis*)], *Streptococcus bovis* [ATCC 49147 (*S. bovis*)], *Escherichia coli* [ATCC 25922 (*E. coli*)], *Pseudomonas aeruginosa* (clinical isolate), *Shigella sonnei* [ATCC 25931 (*Sh. sonnei*)], *Klebsiella pneumoniae* [ATCC 700603 (*K. pneumoniae*)], and *Salmonella Typhimurium* [ATCC 14028, (*S. Typhimurium*)]. Reaction mixtures (50 μL) were loaded into each well and incubated overnight at 37 ± 2°C. The sensitivity of the test organisms was determined by measuring the diameter of the inhibition zone around each well to the nearest colony (mm).

### Determination of MIC and MBC

The MIC was determined in Mueller Hinton broth using serial two-fold dilutions of AgNPs at concentrations ranging from 5 to 160 mg/mL with normalized bacterial concentrations (1 × 10^8^ CFU/mL, 0.5 McFarland's standard). The positive control contained M-H broth with tested bacterial concentrations, and the negative control contained only inoculated broth. The samples were incubated for 24 h at 37°C. The MIC is the minimum concentration of AgNPs that visually inhibits 99% of bacterial growth. The MBC was determined by inoculating samples that did not show any growth of pathogenic bacteria on Müller-Hinton agar and observing growth after incubation at 37°C for 24 h. The MBC is the minimum concentration yielding no growth.

### Toxicity assay of AgNP nematicidal activity

*P. redivivus* was obtained from the Nematology Lab in the Faculty of Agriculture at Suez Canal University (Ismailia, Egypt). The nematode cultures were maintained on nematode growth agar (peptone 2.5 g/L; 1 M potassium phosphate 25 mL; NaCl 3 g/L; 1 M MgSO_4_.7H_2_O 1 mL; 1 M CaCl_2_.2H_2_O 1 mL; cholesterol 1 mL; agar 17 g/L) that was incubated at 21 ± 2°C in the dark for several weeks. *E. coli* was supplied as a food source. Juvenile nematodes were collected using a Clorox solution (1 M NaOH: 5% NaCl, 5:2). The solution was added to the nematode plate for 15 min and 1 mL of solution was added to 1.5 mL centrifuge tubes. The tubes were centrifuged for 2 min at 2000 rpm, and the supernatant was washed with K-medium (0.032 M KCl, 0.051 M NaCl; Williams and Dusenbery, 1990). The toxicity assay was carried out on nematode agar plates with AgNP suspensions. *P. redivivus* (1 × 10^3^ mL^−1^) was exposed to 0, 50, 100, 150, or 200 μg/mL of AgNPs in a 5 mL nematode suspension to determine estimated mortality (%) and survival number. Tests were performed in quadruplicate. The exposure periods for the assays were 24, 36, and 48 h. The samples were analyzed using visual microscopy.

### Statistical analysis

Data were analyzed using ANOVA by using SAS statistical software (SAS Institute, Cary, NC, USA). When the main effect was significant (*P* < 0.05), differences between means were evaluated for significance by using Duncan's multiple-range test.

## Results and discussion

**Figure 1:**
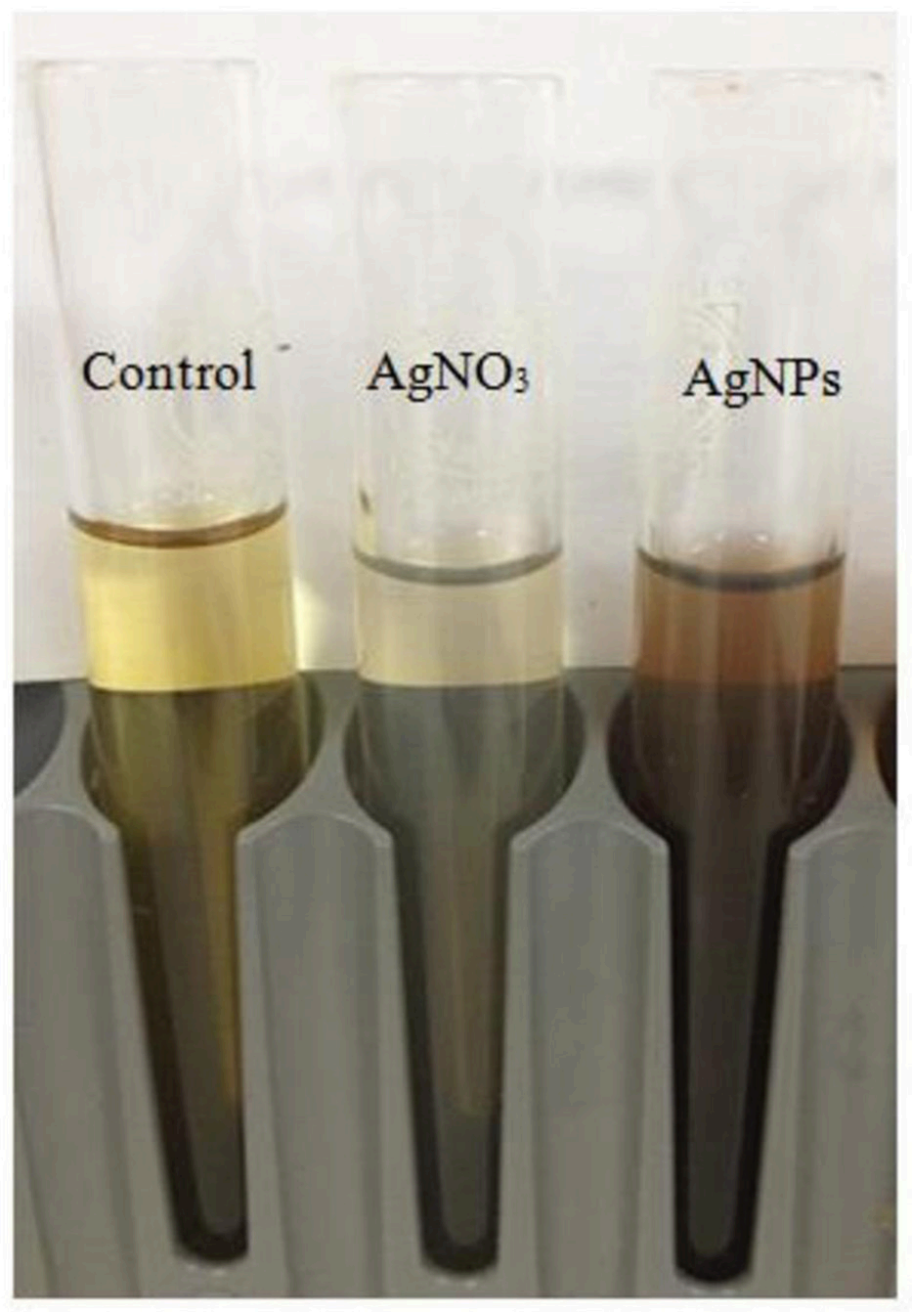
**Color changes in the supernatant of free bacterial cells (*Bacillus pumilus*) after addition of silver nitrate for nanoparticle (AgNP) synthesis**.

The physical properties of biologically synthesized AgNPs may vary depending on the type of microorganism used for synthesis (Ahmad et al., 2003). In this study, about 20 bacterial isolates were screened for their ability to synthesize regularly-shaped silver nanoparticles. The 20 morphologically distinct bacterial isolates were grown on agar plates. The change of the cell-free filtrate from yellow to brown was the primary assay to determine whether the isolates that were capable of synthesizing AgNPs (Figure 1).

**Figure 2:**
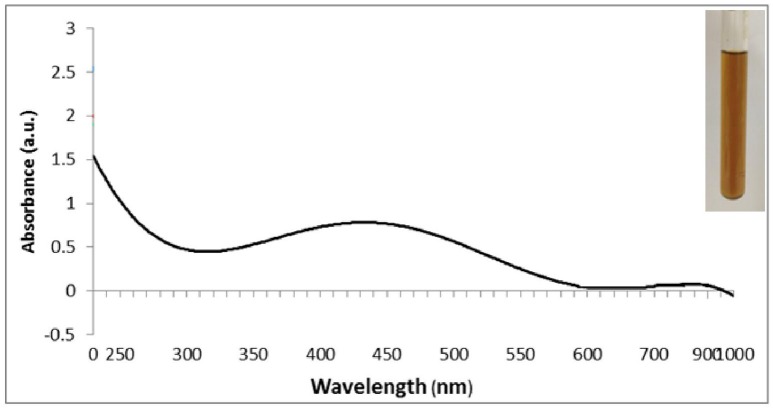
**UV–visible spectrum of silver nanoparticles (1 mM aqueous solution of AgNO_3_) synthesized by *Bacillus pumilus***. The inset of the figure shows a test tube of the silver nanoparticle solution formed at the end of the reaction.

**Figure 3:**
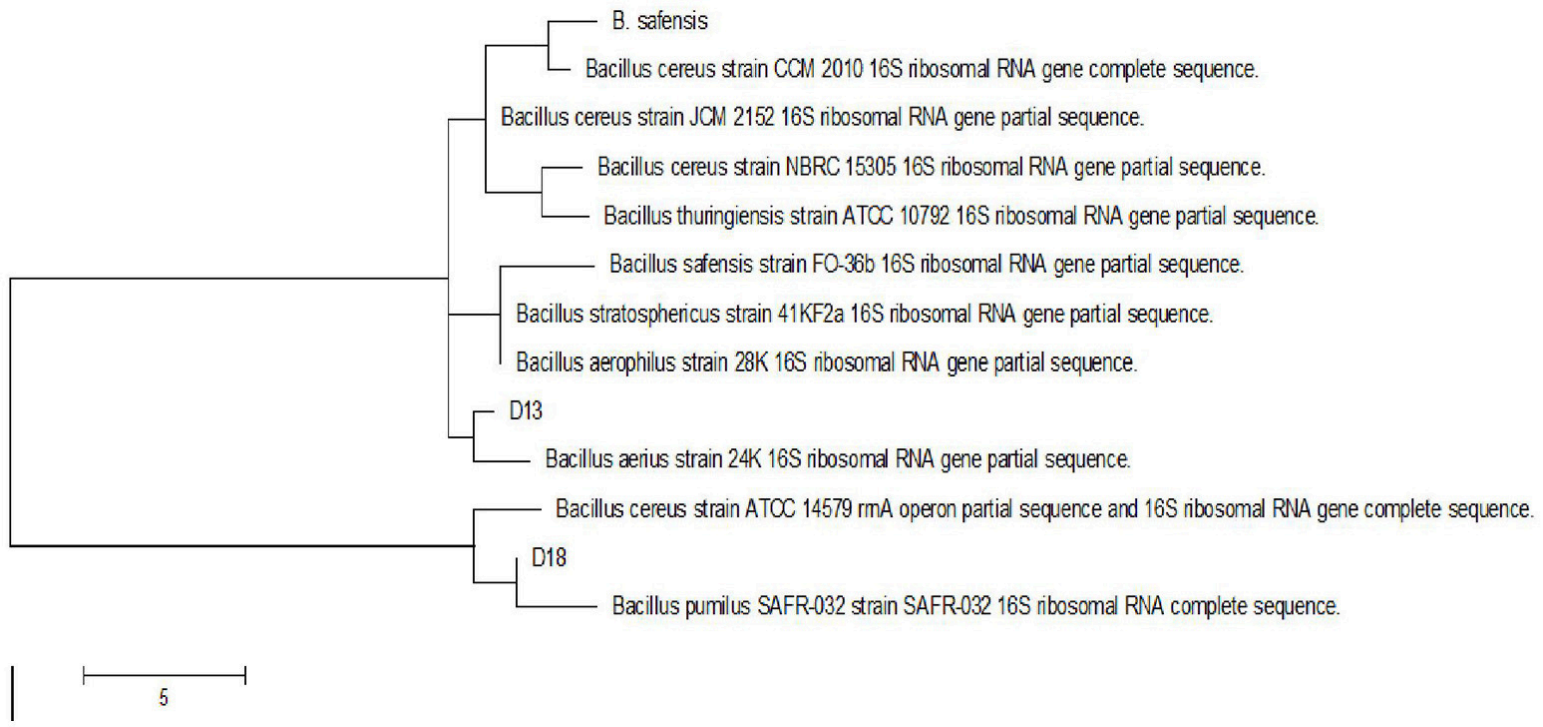
**Phylogenetic tree of *Bacillus* isolates (D18) based on partial 16S rRNA sequences**.

Following the addition of silver nitrate (1 mM) to the filtrate of bacterial cultures, only the D18 isolate was able to change the color from yellowish to dark-brown, at 60°C within 2 h (Figure 1). The alteration in color is based on the surface plasmon resonance (SPR) of AgNPs. The control and blank tubes remained colorless (Chaudhari et al., 2012; Kirubha and Alagumuthu, 2013; Yamal et al., 2013). We validated AgNP synthesis using UV-visible spectrophotometry (Figure 2). This type of scanning has been useful in gauging SPR absorption of silver nanoparticles (Shahverdi et al., 2007; Ansari et al., 2011; Singh et al., 2011; Ponarulselvam et al., 2012). A strong SPR band was only observed at 430 nm for the D18 isolate. The UV-visible spectral bands observed at 430 nm are characteristic of silver nanoparticles (Figure 2; Petit et al., 1993; Ahmad et al., 2003; Singaravelu et al., 2007). Subsequently, the D18 isolate was identified as *B. pumilus* using 16S rRNA sequencing (Figure 3).

**Figure 4:**
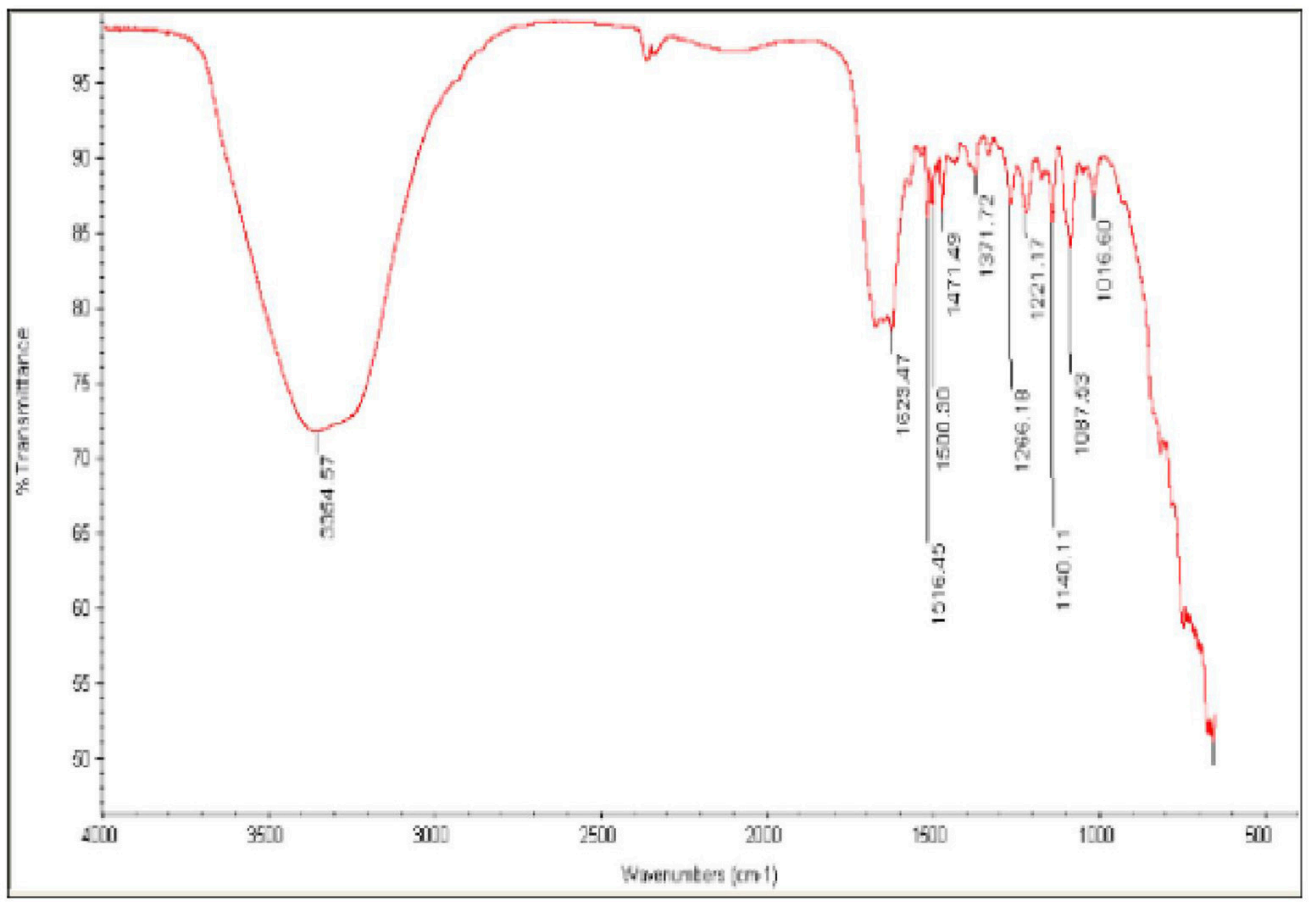
**Fourier-transform infrared (FT-IR) spectrum of silver nanoparticles synthesized by *B. pumilus***.

Fourier transform infrared spectroscopy (FT-IR) measurements were used to determine if the biomolecules are susceptible to Ag^+^ ion reduction and identify the capping agent of the bioreduced AgNPs (Figure 4). A band at 3500–3000 cm^−1^ in the FT-IR spectra is specific to the extending vibration of primary amines. The appearance of a band at about 1743 cm^−1^, which is assigned to extension of C = O vibrations in carboxylic acids, aldehydes, and ketones, was notable and suggests the oxidation of the hydroxyl groups in bacterial hydrolysates coupled to the reduction of silver ions (Saha et al., 2010). The bands observed in the FT-IR spectra at 1650 cm^−1^ are an indicator of linkages of amide I and II (Sharma et al., 2012). The vibrations at 1500–1000 cm^−1^ may indicate methylene scissoring vibrations from the proteins in the bacterial filtrate (Sharma et al., 2012). The FT-IR spectra suggest that protein is the capping agent responsible for enclosing the bio-AgNPs (Vigneshwaran et al., 2007).

**Figure 5:**
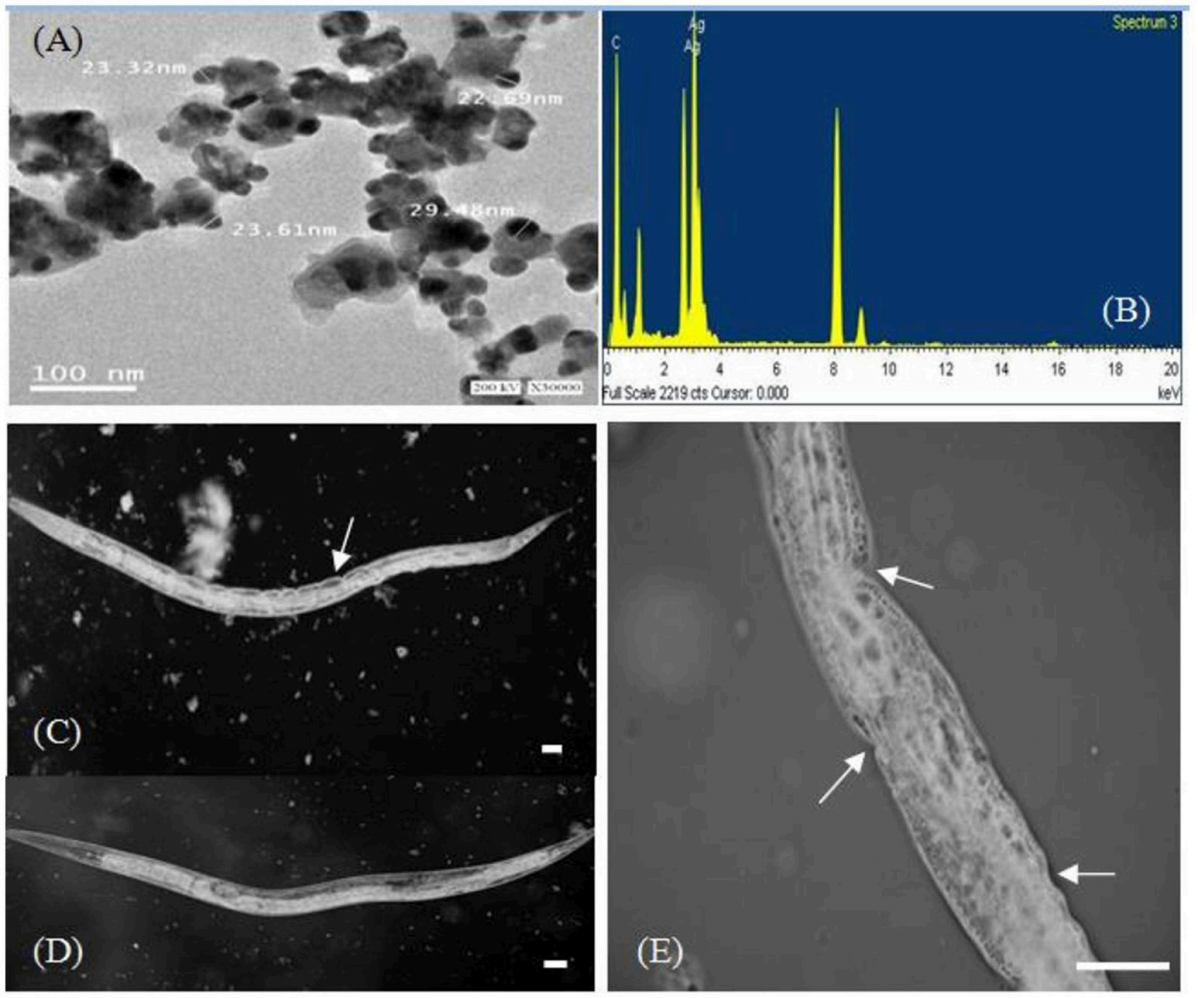
**TEM micrograph at 30,000 × magnification (A)** and energy-dispersive spectroscopy spectrum **(B)** of silver nanoparticles synthesized by *B. pumilus*. High-resolution microscopic images of the nematode *P. redivivus* **(C,E)** showing the epidermal malformation and necrosis (white arrows) resulting from AgNPs compared with controls **(D**; bar = 300μm).

The morphology of the silver nanoparticles was analyzed using TEM (Figure 5A). The TEM images show mediated silver nano powder has triangular, hexagonal, spherical, pseudospherical, and some undefined morphology, with traces of agglomeration due to the binding of the biological molecules to the nanoparticles present in the bacteria (Malarkodi and Annadurai, 2013). The diameter of the nanoparticles ranged from 22.69 to 29.48 nm.

We successfully used bacterial culture to synthesize small, relatively uniform, extracellular silver nanoparticles. Energy-dispersive spectroscopy was used to confirm the presence of elemental silver (Figure 5B), and silver nanocrystals were verified by an optical absorption band peak at 3 KeV, which is the same absorption as metallic silver nanocrystals based on SPR (Magudapathy et al., 2001). Other EDX signals emitted from O, N, and C atoms were also observed. These peaks are probably due to X-ray emissions from the proteins and enzymes present in the cell free filtrates (Das et al., 2010). It is well-known that proteins can bind to nanoparticles either through free amino groups or through cysteine remnants in the protein (Mandal et al., 2005). Analysis of elemental composition via EDX showed the presence of silver atoms produced by *B. pumilus*; these results agree with the TEM results (Figures 5A,B).

The effects of AgNPs on a nematode cuticle (*P. redivivus*) were observed using high-resolution microscopy. Figures 5C,E shows that the nematode cuticle “burst” when treated with AgNPs at 50, 100, 150, and 200 μg/mL after 48 h (Figures 5C,E); meanwhile, there was no effect on the control (Figure 5D). The same result was observed by Samberg et al. (2010), who found that AgNPs could penetrate animal skin and cause inflammation and edema at elevated levels, as well as epidermal hyperplasia at the highest concentration. Additionally, Wu et al. (2009) observed epidermal damage in various animal tissues subjected to AgNP treatment. Our results are in agreement with many previous studies (Vijver et al., 2003; Cunha et al., 2011; Kim et al., 2012).

**Figure 6:**
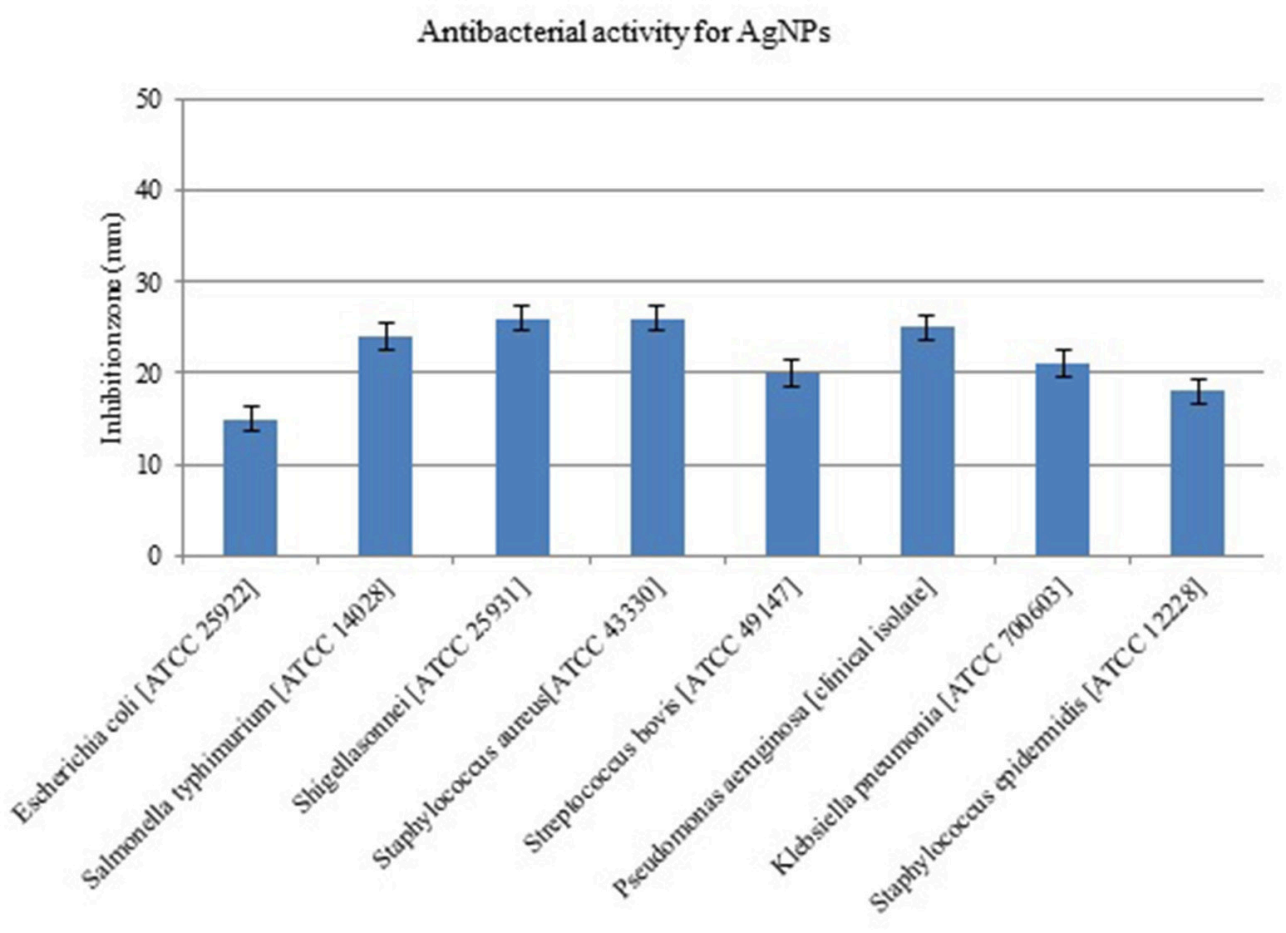
**Antimicrobial activity of silver nanoparticles (AgNPs) synthesized by *B. pumilus* against different pathogenic bacterial strains**.

The AgNPs formed by *B. pumilus* strain exhibited antibacterial activity against *E. coli, S. bovis, K. pneumoniae, S. Typhimurium, P. aeruginosa, S. sonnei*, and *S. aureus* (Figure 6). Shrivastava et al. (2007) suggested that Gram-positive bacteria are more sensitive to silver nanoparticles than Gram-negative bacteria because of the interactions of positively charged silver nanoparticles with negatively charged lipopolysaccharides. Yamanaka et al. (2005) reported antimicrobial effects of AgNPs on *S. aureus* and *E. coli*. The effect of AgNPs on bacterial strains may be due to the release of their ions inside the bacterial cell (Feng et al., 2000; Morones et al., 2005). The MIC and MBC of biosynthesized AgNPs for the majority of evaluated strains were in the range 5–20 μg/mL; meanwhile, for *E. coli* the MIC and MBC were ~40 and 80 μg/mL, respectively (Table 1). The three Gram-positive pathogens had MIC values of 5 μg/mL; however, the MIC for the Gram-negative strains was 10 μg/mL. The MBC did not exceed 20 μg/mL for any strain. Our results show better antibacterial activity than those of Ansari et al. (2011), Ayala-Núñez et al. (2009), and Fernández et al. (2008), who reported MIC and MBC values ranging from 12.5 to 25 μg/mL. This was likely due to the smaller size of the nanoparticles in our study. Li et al. (2011) showed complete inhibition of the growth of *S. aureus* ATCC 6538P at 20 μg/mL. The mechanism(s) of AgNP antimicrobial activity may differ depending on the bacterial species and the nanoparticle size (Ansari et al., 2011).

**Table 1:** **The mean of MIC and MBC values for silver nanoparticles (AgNPs) tested using human pathogens in M-H broth**.

**Organism**	**AgNPs**		**Tetracycline**	
	**MIC (μg/mL)**	**MBC (μg/mL)**	**MIC (μg/mL)**	**MBC (μg/mL)**
*Escherichia coli* ATCC 25922	40±1.7	80±3.0	10±0.0	20±0.05
*Pseudomonas aeruginosa* (clinical isolate)	10±0.0	20±3.6	10±1.3	20±1.0
*Salmonella Typhimurium* ATCC 14028	10±0.58	10±0.46	10±0.0	20±0.0
*Klebsiella pneumoniae* ATCC 700603	10±0.0	10±1.73	5±0.0	20±0.0
*Shigella sonnei* ATCC 25931	10±1.0	10±0.5	5±0.0	10±1.0
*Streptococcus bovis* ATCC 49147	5±0.0	10±0.1	10±3.4	20±0.0
*Staphylococcus epidermidis* ATCC 12228	5±0.0	10±0.5	10±0.7	10±0.0
*Staphylococcus aureus* MRSA ATCC 43330	5±1.3	20±1.0	20±0.0	<20±0.23

Determination of MICs and MBCs for AgNPs was performed at least three times in duplicate. MIC, Minimal inhibitory concentration; MBC, Minimal bactericidal concentration.

**Figure 7:**
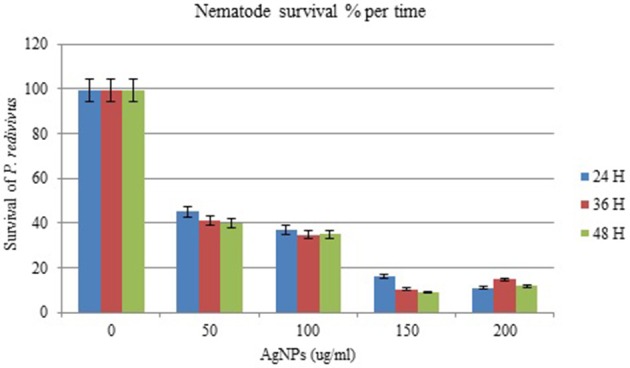
**The effect of different concentrations of silver nanoparticles (AgNPs) synthesized by *B. pumilus* on *P. redivivus* survival (%) after 24, 36, and 48 h incubation in nematode growth medium**.

The effect of AgNPs on the survival and mortality (shown as % per time) of *P. redivivus* was significant at 50, 100, 150, and 200 μg/mL compared to untreated nematodes (Table 2). Moreover, increased exposure time was proportional to mortality (%) for all except the two highest concentrations (150 and 200 μg/mL). This was likely due to the aggregation of AgNPs at these high concentrations. This result agrees with those of Kim et al. (2012), Baek and An (2011), and Lee et al. (2011), who all found that the aggregation of nanoparticles at high concentrations inhibited nanoparticle toxicity. In addition, nematode survivability increased significantly with decreased exposure time (Figure 7). We believe that increasing exposure time may increase the chances of nematode feeding, interactions with AgNPs, and toxicity. This is supported by Meyer et al. (2010), who confirmed the presence of AgNPs in the nematode gut and unlaid eggs. Furthermore, Chithrani and Chan (2007) reported that the cellular uptake of nanoparticles is controlled by the wrapping time of binding sites on the cell surface.

**Table 2:** **The effect of different concentrations of silver nanoparticles (AgNPs, synthesized by *B. pumilus*) on *P. redivivus* after 24, 36, and 48 h of nematode growth**.

**AgNPs (μg/mL)**	**Mortality (%)**		
	**24 h**	**36 h**	**48 h**
Untreated	0.50^d^	0.500^d^	0.63^c^
50	55.0^c^	58.00^c^	60.0^b^
100	63.0^b^	65.00^b^	65.33^b^
150	89.0^a^	89.67^a^	91.0^a^
200	84.0^a^	85.17^a^	88.0^a^

Means in each column followed by the same letter are not significantly different (P < 0.05) as determined by Duncan's multiple range test.

## Conclusions

Bionanotechnology contributes to the development of therapeutic interventions for many conditions, including cancer, bacterial infections, and viruses. Biosynthesis of nanoparticles eliminates many problems associated with chemical synthesis. Biosynthesized nanoparticles can be used in minimal amounts to inhibit pathogen and nematode growth without any harmful side-effects, thus replacing conventional antibiotics and chemical nematicides. Nanoparticle production is ecofriendly, does not require any solvents or toxic chemicals, and is easily scalable. We have shown that AgNPs can be used as potent antibacterial agents against various pathogenic microorganisms.

## Author contributions

WM, TA, and AE conceived and designed the experiments; AE carried out the antimicrobial activities studies; TA performed the nematicidal experiments; TA and AE analyzed the data; WM, TA, and AE wrote the paper. All authors have read and approved the final manuscript.

### Conflict of interest statement

The authors declare that the research was conducted in the absence of any commercial or financial relationships that could be construed as a potential conflict of interest.
